# The Capiat

**Published:** 1891-11

**Authors:** 


### THE CAPIAT.

We publish in this issue a description and cut of this unique instrument; the invention of a Texas physician. In this instance, as in many others, "necessity" was "the mother of (the) invention." Dr. Poynor says: "Any man who has ever tried to seize with his fingers the slippery contents of a contracted or contracting womb, will appreciate both the difficulty of doing so satisfactorily, and the need of some instrument to facilitate their removal." In our judgment, it is an instrument of much merit; and without having had any experience in its use, we would suppose that it is admirably adapted to the purpose for which it was made. It is an ingenious piece of mechanism, and its contrivance reflects much credit upon the doctor. He and many other Texas physicians have used it, and are satisfied with its work. A moment's reflection will convince any one of the important part it is called upon to play in abortions; and the dangers resulting from a neglect or inability to thoroughly evacuate the womb of all contents before leaving the patient.

We invite, at the hands of our readers, a careful examination of the Capiat, and are satisfied they will find it a great help to them, as it has been to its inventor, in practice. It is for sale by L. Ohlson, the manufacturer, at 220 Main street, Dallas, Texas.

